# Characterization of eukaryotic DNA *N*^6^-methyladenine by a highly sensitive restriction enzyme-assisted sequencing

**DOI:** 10.1038/ncomms11301

**Published:** 2016-04-15

**Authors:** Guan-Zheng Luo, Fang Wang, Xiaocheng Weng, Kai Chen, Ziyang Hao, Miao Yu, Xin Deng, Jianzhao Liu, Chuan He

**Affiliations:** 1 Department of Chemistry, Institute for Biophysical Dynamics, Department of Biochemistry and Molecular Biology, Howard Hughes Medical Institute, The University of Chicago, 929 East 57th Street, Chicago, Illinois 60637, USA; 2 Wuhan University School of Pharmaceutical Sciences, Wuhan 430071, China; 3 College of Chemistry and Molecular Sciences, Key Laboratory of Biomedical Polymers of Ministry of Education, Wuhan University, Wuhan 430072, China

## Abstract

Although extensively studied in prokaryotes, the prevalence and significance of DNA *N*^6^-methyladenine (6mA or m^6^dA) in eukaryotes had been underappreciated until recent studies, which have demonstrated that 6mA regulates gene expression as a potential heritable mark. To interrogate 6mA sites at single-base resolution, we report DA-6mA-seq (DpnI-Assisted *N*^6^-methylAdenine sequencing), an approach that uses DpnI to cleave methylated adenine sites in duplex DNA. We find that DpnI cuts other sequence motifs besides the canonical GATC restriction sites, thereby expanding the utility of this method. DA-6mA-seq achieves higher sensitivity with nanograms of input DNA and lower sequencing depth than conventional approaches. We study 6mA at base resolution in the *Chlamydomonas* genome and apply the new method to two other eukaryotic organisms, *Plasmodium* and *Penicillium*. Combined with conventional approaches, our method further shows that most 6mA sites are fully methylated on both strands of DNA at various sequence contexts.

## Abstract (web summary)

DNA *N* ^6^-methyladenine is prevalent in prokaryotes, and is recently also detected in eukaryotes such as roundworm and fly. Here, Luo *et al*. report a DpnI-assisted base resolution method that detects 6mA genome-wide with nanograms of input DNA and lower sequencing depth than the previous restriction enzyme-based approach.

## Graphical abstract

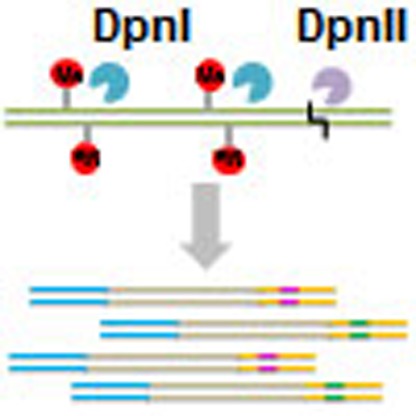

DNA *N*^6^-methyladenine (6mA) is the most prevalent DNA modification in prokaryotes but rare in eukaryotes^1^,^2^. Only certain unicellular eukaryotic organisms, such as the green algae *Chlamydomonas*^3^ and the ciliate *Tetrahymena*^4^, have been known to possess detectable 6mA in the genomic DNA previously^5^. Benefiting from the latest developments of highly sensitive detection methods, recent studies have revealed the presence of 6mA in metazoans *C. elegans*^6^ and *Drosophila*^7^ in additional to green algae^8^,^9^, as well as potential 6mA methyltransferases and demethylases, raising the possibility that 6mA may be more prevalent in eukaryotes^1^,^10^. A latest study further reports discoveries of 6mA, albeit in very low abundances, in the genomes of vertebrates, highlighting the prevalence of 6mA in more extensive eukaryotes^11^.

Besides modern mass spectrometry that can accurately quantify the amount of DNA modifications, next-generation sequencing (NGS) provides another powerful tool to gain crucial genome-wide distribution information of these DNA modifications^12^,^13^. 6mA specific antibodies have been used to recognize and enrich 6mA-containing DNA fragments for sequencing studies^6^,^7^. 6mA can be profiled by coupling immunoprecipitation (IP) with NGS. A quantitative map at single-base resolution with the modification percentage information is more desirable, however. Unfortunately, a bisulfite-treatment equivalent of deamination approach has yet to be developed for the sequencing of methylated adenines. Although single-molecule real-time (SMRT) sequencing can distinguish 6mA from A and has been successfully applied in prokaryotes containing small-sized genomes^14^,^15^, the relatively high expense and low throughput have prevented its wide applications in eukaryotic organisms with much larger genomes.

Restriction–modification systems are widely adopted in bacteria to resist phage infections^16^. Restriction enzymes specifically digest invading phage DNA while the host DNA is modified and resists digestion. In the co-evolutionary arm race between phages and bacteria, certain phages have evolved to modify their own DNA to escape restriction enzyme-based digestion; in response, certain bacteria have evolved to attack modified phage DNA^17^,^18^,^19^. Although restriction–modification systems are not thought to exist in eukaryotes, collections of various restriction enzymes provide invaluable toolkits for the study of DNA modifications in prokaryotic and eukaryotic genomes^20^,^21^. In our recent work, we reported a restriction enzyme-assisted sequencing approach to identify single 6mA sites in the genome of *Chlamydomonas*^8^. Two restriction enzymes, CviAII and DpnII, were introduced which cleave DNA at CATG and GATC sites, respectively, leaving methylated sites C(6mA)TG and G(6mA)TC intact^22^,^23^. However, these two enzymes cannot distinguish fully methylated (symmetric methylation on double-stranded DNA) or hemi-methylated sites (only one strand is methylated), and incomplete digestion may generate false-positive methylation assignments because of the dominance of unmethylated restriction sites^8^. To control the false-positive rate, a minimal 10 × whole-genome coverage of sequencing depth is usually necessary, leading to high costs in studies of large genomes.

In the current study, we show that a 6mA-sensitive restriction enzyme, DpnI, preferentially cleaves duplex DNA at fully methylated sites^24^. With reduced sequencing depth, a new approach using DpnI obtains the equivalent 6mA map of *Chlamydomonas* at single-base resolution. Importantly, the new method requires much less starting material (as low as 10 ng) and reduced sequencing cost, but achieves higher sensitivity, making it particularly suitable for characterizations of sites with relatively low 6mA abundances and processes with limited materials. In addition, DpnI not only recognizes a canonical GATC sequence motif, but also cleaves fully methylated double-stranded DNA at CATC/GATG sites (CATC and GATG are complementary to each other), which further expands the application scope of this method. By combining results using this new approach with previous data, we conclude that most 6mA sites in *Chlamydomonas* are fully methylated at various sequence contexts, further supporting the role of 6mA as an epigenetic mark in eukaryotes^9^. This method is particularly good for the study of low abundance 6mA sites because of its high sensitivity. We apply the method to two other eukaryotic organisms with relatively low 6mA abundance in genomic DNA (*Plasmodium falciparum* and *Penicillium chrysogenum*), and characterize 6mA distributions in the genomes of these organisms.

## Results

### Genome-wide identification of 6mA at single-base resolution

**Figure 1:**
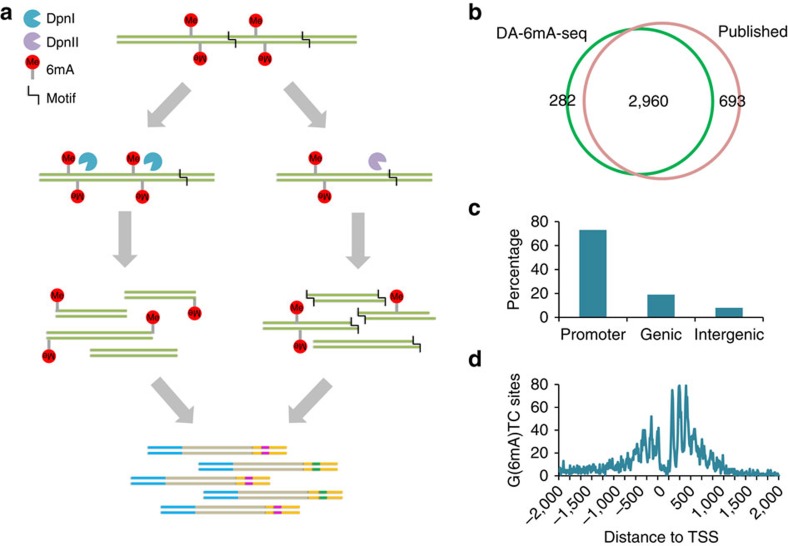
Detection of 6mA by DA-6mA-seq. (**a**) Flowchart of DA-6mA-seq. DpnI cleaves fully methylated G(6mA)TC sites, whereas the cleavage of DpnII is hindered by hemi- or fully-methylated 6mA. After treatment with restriction enzyme, DNA segments are further sheared by sonication to ∼300 bp followed by standard Illumina DNA library construction procedures. (**b**) DA-6mA-seq identifies consistent 6mA sites as reported previously^8^. (**c**) The genomic distribution of 6mA sites in promoter, genic and intergenic regions. Promoter is defined as −1,000 to +1,000 bp region around transcription start sites (TSS). (**d**) The periodic distribution pattern of base-resolution 6mA sites identified by DA-6mA-seq around TSS.

Because DpnI is known to cut methylated G(6mA)TC, we envisioned a restriction enzyme-assisted, high-throughput sequencing approach to identify single 6mA sites genome-wide (Fig. 1a) and validated it using *Chlamydomonas*. Genomic DNA from log-phase sample cultured under constant light was extracted and purified. After treatment with DpnI, long DNA fragments were sonicated to ∼300 bp in length, and then a standard Illumina DNA library was constructed for NGS sequencing. After testing different amounts of input DNA, we were able to use as low as 10 ng input DNA for the digestion step and subsequent library construction (Supplementary Fig. 1). The G(6mA)TC sites should be cleaved by DpnI, and represented as the reads ends in the sequencing output. We developed a bioinformatics pipeline to interrogate the methylation status of each A at GATC context. To overcome the false-positive sites generated by random shearing, a binomial probability distribution was used to determine the cutoff of minimum read ends at GATC sites where the A site could be identified as 6mA while maintaining a false-positive rate below 1%; therefore, <1% of identified 6mA sites might be erroneously assigned. A parallel library was also constructed by using DpnII, which specifically cleaves unmethylated GATC sites (Fig. 1a).

In total, we uncovered 92% GATC sites genome-wide, among which 2.3% were identified as methylated. We compared the identified sites to the 6mA map determined using the DpnII-assisted approach as described previously^8^, and found that at least 91% of sites agreed in two data sets (Fig. 1b). By aligning the single 6mA sites to gene loci, we found that >70% of these 6mA sites are located at promoters defined as −1 kb to +1 kb regions around transcription start sites (Fig. 1c). The periodic distribution pattern (Fig. 1d), a key feature of 6mA distribution associated with nucleosome positioning in the *Chlamydomonas* genome, was recapitulated^8^.

### DpnI recognizes CATC and GATG besides GATC

**Figure 2:**
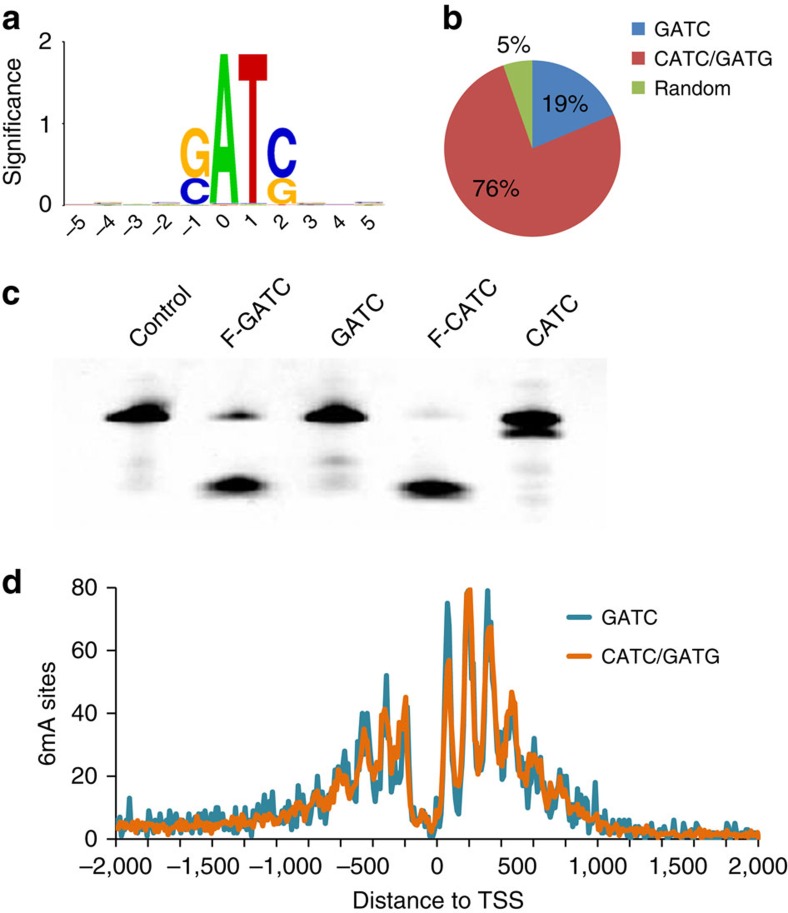
Non-GATC 6mA sites in the *Chlamydomonas* genome identified by DA-6mA-seq. (**a**) The accumulated nucleotide composition of all potential 6mA sites identified by DA-6mA-seq and computation. Sequence logo is generated by WebLogo (ref. 40). The height represents the significance of enrichment of each nucleotide at that position. (**b**) Percentages of 6mA sites at different tetramer sequence contexts. (**c**) DpnI effectively cleaves the fully methylated GATC (F-GATC) and CATC (F-CATC) sites but does not digest unmethylated sites (GATC, CATC). Probes (5 pmol) were treated with DpnI (10 units) overnight at 37 °C. (**d**) The periodic distribution pattern of methylated CATC/GATG sites around transcription start sites (TSS) is nearly identical to the pattern of methylated GATC sites.

DpnI efficiently cleaves G(6mA)TC sites based on previous reportes^22^,^24^. However, our sequencing results have revealed cleavage of a large fraction of methylated non-GATC sites (Fig. 2a). Specifically, 76% of the identified 6mA sites are located in the CATC/GATG context while only 19% are located in the GATC context (Fig. 2b). This high proportion of non-GATC 6mA sites prompted us to confirm the cleavage of C(6mA)TC by DpnI in addition to G(6mA)TC. In fact, a previous report suggested that DpnI could also recognize motifs other than GATC (ref. 25). We synthesized oligonucleotides with 6mA site in the CATC/GATG context, and annealed them to form double-stranded DNA. After treatment with DpnI, the DNA probes with C(6mA)TC and G(6mA)TG motifs were explicitly cleaved at the 6mA site after 12 h of incubation (Fig. 2c). As negative controls, double-stranded DNA probes containing unmethylated GATC or CATC were resistant to cleavage even with overnight incubation. Therefore, the non-GATC sites identified by DA-6mA-seq should be genuine 6mA sites. Indeed, these sites overlap very well with the previous 6mA map determined by using IP followed by sequencing (Supplementary Fig. 2a)^8^. We then inspected the genomic distribution of these non-GATC sites, and found that they were highly enriched at promoter regions (Supplementary Fig. 2b). Furthermore, these 6mA sites form a clear periodic pattern around transcription start sites, which is almost identical to the pattern of G(6mA)TC in the same genomic DNA (Fig. 2d).

The remaining 5% of the 6mA sites are comprised of random sites (Supplementary Fig. 2c). We have previously identified C(6mA)TG sites in the *Chlamydomonas* genome^8^; DA-6mA-seq failed to identify these sites, however, showing the high specificity of DpnI to 6mA-methylated GATC and CATC/GATG sequences, which is consistent with the crystal structure of DpnI bound to duplex DNA^25^,^26^.

About 1.8% of all CATC/GATG sites were detected to be methylated across the *Chlamydomonas* genome, which is analogous to the percentage of 6mA in the GATC context. The methylated adenines occupy only a small fraction of all motifs in the genome, ruling out a possible methylation mechanism similar to that observed in bacteria, which methylates target loci based on the restriction sequence motif. On the other hand, the percentages of the methylated sites in these sequence contexts that we identified are consistently higher than random (6mA accounts for ∼0.4% of the total adenines in the genome^8^), suggesting that these ApT centred sequences are favourably recognized by the methylation machinery.

### 6mA sites are mainly fully methylated in *Chlamydomonas*

**Figure 3:**
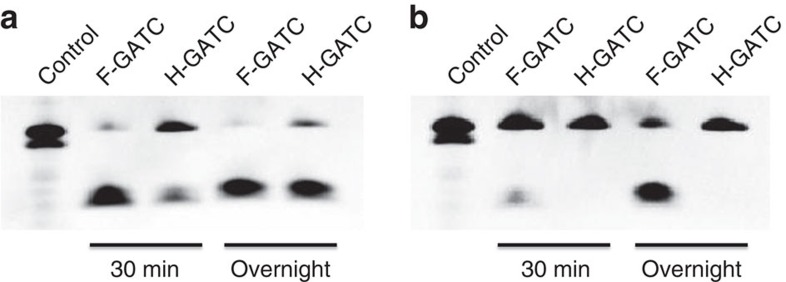
DpnI cleavage assay on fully- or hemi-methylated GATC and CATC/GATG DNA probes. The PAGE gel shows the formation of digested products. (**a**) DNA probes containing fully methylated GATC (F-GATC, 10 pmol) or hemi-methylated GATC (H-GATC, 10 pmol) were treated with DpnI (10 units) for 30 min or overnight at 37 °C. (**b**) DNA probes containing fully methylated CATC/GATG (F-CATC, 10 pmol) or hemi-methylated CATC/GATG (H-CATC, 10 pmol) were treated with DpnI for 30 min or overnight. All sequences used are listed in Supplementary Table 1.

The previous approach using CviAII- or DpnII-assisted digestion cannot distinguish fully methylated and hemi-methylated sites since these two enzymes are hindered by both fully- or hemi- methylation^8^. To explore the sensitivity of DpnI to different methylation status, we synthesized single-stranded DNA probes with or without 6mA at specific sequence motifs, and annealed them to afford double-stranded DNA representing fully and hemi-methylated probes. Consistent with the previous report^22^, DpnI cleaves fully methylated DNA much faster than hemi-methylated DNA containing the GATC motif (Fig. 3a). Specifically, DpnI barely cleaved hemi-methylated CATC/GATG sites, even if the reaction time was excessively prolonged; fully methylated CATC/GATG sites were cleaved by overnight incubation, however (Fig. 3b). We treated the same batch of DNA with DpnI for 30 min or 12 h (overnight), and performed DA-6mA-seq in parallel. Both G(6mA)TC and C(6mA)TC/G(6mA)TG sites in the two data sets concurred (>90% overlapping, Supplementary Fig. 3). These results suggest that the vast majority of the 6mA sites identified by DA-6mA-seq are fully methylated. The additional ∼500 6mA sites that were identified only by using DpnII-based digestion are most likely hemi-methylated^8^. Genome-wide analysis showed no preferences of these sites to promoters, exons or introns (Supplementary Fig. 3), and the abundances at each sites are low, which may indicate aberrant methylation or roles yet to be identified.

### Application of DA-6mA-seq to other organisms

**Figure 4:**
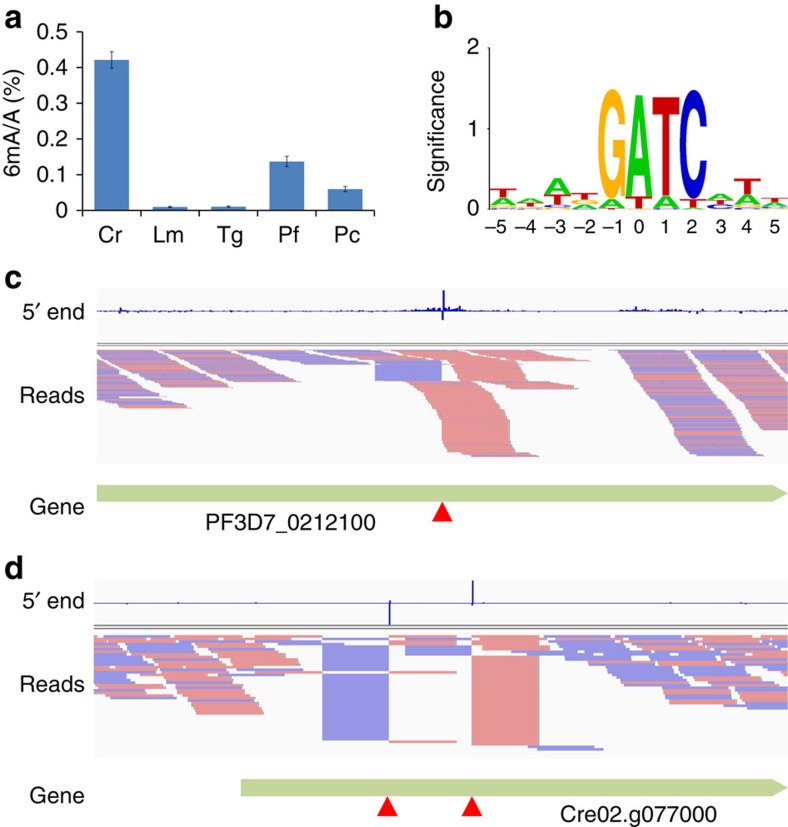
DA-6mA-seq is a highly sensitive method to detect 6mA at specific sites. (**a**) Quantification of 6mA abundances in *Chlamydomonas reinhardtii* (Cr), *Leishmania major* (Lm), *Toxoplasma gondii* (Tg), *Plasmodium falciparum* (Pf) and *Penicillium chrysogenum* (Pc) by LC–MS/MS. Error bars are calculated as the s.d. of three biological replicates. (**b**) The accumulated nucleotide composition of all the potential 6mA sites identified by DA-6mA-seq in *Plasmodium*. Sequence logo is generated by WebLogo (ref. 40). (**c**) The snapshot of genome browser (IGV (ref. 41)) representing a partially methylated site in *Plasmodium*. The top track shows counts of 5′ ends in the selected region. The ‘Reads' track shows aligned reads. Blue segments represent reads mapped to minus strand and red segments represent reads mapped to plus strand. The red triangle marks GATC site. (**d**) The snapshot of genome browser representing two completely methylated sites in *Chlamydomonas*.

Previous studies indicate the presence of 6mA in more extensive eukaryotes^5^,^10^,^27^,^28^,^29^,^30^, including the human malarial parasite *Plasmodium*^5^ and the fungi *Penicillium*^31^. To validate the existence of 6mA in these other organisms, we employed liquid chromatography coupled with tandem mass spectrometry (LC–MS/MS) to quantify the 6mA abundance in four candidate organisms: *Leishmania major*, *Toxoplasma gondii*, *Plasmodium falciparum* and *Penicillium chrysogenum*^8^,^32^,^33^ (Fig. 4a); the genomes of *Plasmodium* and *Penicillium* were found to contain relatively abundant 6mA (∼0.05–0.15% 6mA/A). We then applied DA-6mA-seq to map 6mA in the genomes of these two organisms. Surprisingly, the distribution patterns of 6mA in the genomes of *Plasmodium* and *Penicillium* are very different from that in *Chlamydomonas*. In *Plasmodium*, ∼1/5 of GATC sites are extensively methylated all across the genome with methylated CATC or GATG detected at very low level (<1%; Fig. 4b). The extensive G(6mA)TC sites identified are inconsistent with the gross 6mA abundance of the genome. We reasoned that the methylation level of each site might be relatively low; in other words, only a small proportion of individual cells contain 6mA at specific sites, whereas other cells do not. To validate this speculation, we re-analysed the sequencing data and considered the randomly sheared reads overlapping with each GATC sites. If random reads are more than enzyme-digested reads, we defined the site as partially methylated (Fig. 4c). The ratio of random reads to enzyme-digested reads can also help to estimate the percentage of methylation at each specific site^8^. Under this definition, the majority of G(6mA)TC sites (97%) were methylated with a methylation percentage below 10% in *Plasmodium* (Supplementary Fig. 4a); in contrast, only 9% of G(6mA)TC sites were partially methylated in the *Chlamydomonas* genome, which is consistent with much lower 6mA abundance measured in the genome of *Plasmodium* than that in *Chlamydomonas* (Fig. 4d and Supplementary Fig. 4a). The wide-spread G(6mA)TC sites are distributed randomly across the genome without any enrichment to gene context (Supplementary Fig. 4b).

Far fewer 6mA sites were detected in the genome of *Penicillium*, corresponding to even lower 6mA abundances at individual sites. Similar to *Plasmodium*, most of the G(6mA)TC sites were partially methylated with a low percentage of methylation ratio (Supplementary Fig. 4a). In comparison, the DpnII-assisted approach detected very limited G(6mA)TC sites (∼100 in *Plasmodium* and ∼20 in *Penicillium*) in these two genomes (Supplementary Fig. 4c), presumably because of the low sensitivity of the DpnII-assisted approach to detect G(6mA)TC sites with low levels of methylation. The application of DA-6mA-seq in *Plasmodium* and *Penicillium* demonstrated high sensitivity of this method for studying genomes with low 6mA abundance. The uncovered distribution pattern of 6mA in the genomes of *Plasmodium* and *Plasmodium* may suggest complicated roles of 6mA in different organisms.

## Discussion

Recent studies have revealed unique distribution patterns and potential biological significances of 6mA as a new epigenetic DNA mark in eukaryotes^9^. The genome-wide distributions of 6mA have been profiled in the genomes of *C. elegans*^6^, *Drosohpila*^7^ and *Chlamydomonas*^8^; however, the functional roles of 6mA in more extensive organisms remain largely unexplored^10^. IP-based methods require a large amount of input DNA, whereas single-molecule real-time sequencing is restricted to organisms with small genome sizes due to throughput and cost issues. Increasing body of evidence shows the presence of 6mA in early embryos of *Drosohpila*^7^ and vertebrates (unpublished data and correspondences), highlighting the urgency of new effective and economical sequencing methods to carry out large-scale studies. DA-6mA-seq takes advantage of the restriction enzyme, DpnI, which sensitively and specifically cleaves 6mA-containing motifs. DA-6mA-seq requires far fewer sequencing reads to obtain base-resolution information than previous methods, which significantly increases detection accuracy. The optimized procedure reduces the initial DNA amount to 10 ng for standard library construction, while maintaining high sensitivity.

DpnI is known to specifically cleave methylated GATC sites^25^,^26^. Here we found that DpnI not only recognizes the canonical G(6mA)TC sites, but also cleaves C(6mA)TC or G(6mA)TG sites, which further expands the scope of its utility. DpnI could serve as a platform to engineer additional restriction enzymes that recognize other sequence motifs with modification. The wide-spread C(6mA)TC and G(6mA)TG sites in *Chlamydomonas* indicate that palindromic sequence is not a necessary factor to determine the target loci, suggesting a methylation system in eukaryotes distinct from those in prokaryotes^34^.

DpnI only cleaves fully methylated sites in a limited time period; therefore, 6mA sites identified by DA-6mA-seq are symmetrically and fully methylated. In *Chlamydomonas*, the prevalence of symmetric methylated ApT suggests the presence of methyltransferase(s) working on hemi-methylated adenines, which faithfully maintains fully methylated status after DNA replication; it is also a hallmark of eukaryotic 5mC DNA methylation. In prokaryotes, methyltransferases recognize sequence motifs as target sites. We have shown that the majority of 6mA sites in the genome of *Chlamydomonas* occur at the core sequence ApT, without flanking sequence preference. Our results here confirmed the fully methylated nature of the majority of 6mA sites without the pre-requisite palindromic motif in *Chlamydomonas*. All of this evidence further indicates that 6mA is a heritable epigenetic mark in this organism.

Limited by the detection method, the presence of 6mA in many eukaryotic organisms is controversial^1^,^5^,^10^. Mass spectrometry-based methods, although sensitive enough to detect the very low abundance of nucleotide modifications, can only provide the overall ratio of the modification in total DNA. In the case of 6mA, bacterial contaminations could easily confound the mass spectrometry signal. High-throughput sequencing coupled with bioinformatic analysis is a powerful strategy to assure eukaryotic origin and to reveal the distribution pattern of 6mA and other modifications^35^,^36^,^37^,^38^. The DA-6mA-seq presented here is highly sensitive and requires only nanograms of input DNA, making it an ideal method to study low abundance 6mA sites as well as biological processes that yield only limited materials. We applied DA-6mA-seq to two selected eukaryotes, *Plasmodium* and *Penicillium*, both of which possess low 6mA abundances in their genomes. DA-6mA-seq effectively identified thousands of single 6mA sites, which not only confirms the wide-spread presence of 6mA, but also showcases the power of this new approach. DA-6mA-seq offers a highly sensitive, rapid, easy-to-use but low-cost method to investigate 6mA in more uncharacterized organisms or biological processes with limited materials.

## Methods

### DNA materials

*Chlamydomonas* DNA was extracted from log-phase strain CC 1609 cultured in standard tris-acetate-phosphate medium at 25 °C under constant light. Genomic DNA of *Leishmania major*, *Toxoplasma gondii* and *Plasmodium falciparum* were purchased from American Type Culture Collection (ATCC; Cat. no. 30012D, 50174D, PRA-405D, respectively). *Penicillium chrysogenum* DNA was extracted by DNeasy Plant Mini Kit (Qiagen, Cat. no. 69104).

### DA-6mA-seq

Approximately 10 ng purified genomic DNA was digested in 0.5h or 12 h by using 20 U DpnI (NEB, Cat. no. R0176S). Digested DNA segments were further sheared by Bioruptor (Diagenode) to ∼300 bp. After purification by AMPure XP beads (Beckman Coulter, Cat. no. A63880), DNA segments were end-repaired, followed by 3′-adenylation and adaptor ligation according to standard Illumina TruSeq DNA sample preparation procedures. Adaptor-ligated DNA segments were PCR amplified for 15 cycles, purified by AMPure XP beads and suspended in 20 μl of resuspension buffer to yield the sequencing library. Quality control and concentration measurement were performed using Agilent 2100 Bioanalyzer DNA 1000 Chip and qPCR. Library was sequenced by using Illumina HiSeq 2500.

### Data analysis

After adaptor trimming and quality control, reads were aligned to reference genome (Cre4 for *Chlamydomonas*, PlasmoDB-24 for *Plasmodium* and Pench1 for *Penicillium*) by Bowtie^39^. The positions of 5′ ends were analysed genome-wide as the potential DpnI cleavage sites (assigned as *x*). A binominal distribution model was assumed that each read could be randomly sheared and mapped with a probability *P*=1/gs (gs=genome size) or cleaved by DpnI. The total number of reads was assigned as *n*. The *P* value of each genomic locus with 5′ reads ends was calculated as 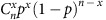. The *P*-values were further corrected by Bonferroni correction, and FDR<0.01 was set as the cutoff to determine a real DpnI cleavage site.

### Restriction enzyme digestion assay

Oligonucleotides containing 6mA were prepared using Applied Biosystems 392 DNA synthesizer with phosphoramidites from Glen Research and purified following standard protocols. Oligonucleotides without 6mA were ordered from IDT. The sequences are listed in Supplementary Table 1. The double-stranded DNA sequences were obtained by annealing single-stranded complementary oligonucleotides. Double-stranded DNA (10 pmol) was digested by 20 U DpnI for 30 min or overnight. After the digestion reaction, the DpnI enzyme was heat-inactivated; then, the product mixture was separated by denaturing electrophoresis using Novex 15% TBE-Urea PAGE gel (Life Technologies, Cat. no. EC6885BOX) and visualized by Bio-Rad Molecular Imager PharosFX system.

### Quantitative analysis of 6mA by LC–MS/MS

Genomic DNA was denatured and digested by nuclease P1 (Sigma-Aldrich, Cat. no. N8630) in 10 mM NH4OAc pH 5.3 at 42 °C overnight. Then, 1 μl of venom Phosphodiesterase I (Sigma-Aldrich, Cat. no. P3243) was added to further digest the DNA sample at 37 °C for 1 h. Finally, NH4HCO3 (1 M, 2 ml) was added which was followed by 1 U of alkaline phosphatase from *E. coli* (Sigma-Aldrich, Cat. no. P5931). Before being uploaded into LC–MS/MS, solution was diluted four times. The ratio of 6mA to A was calculated and normalized based on a standard curve, according to the detailed procedure described by Jia *et al*.^32^.

## Additional information

**Accession codes**: The high-throughput data used in this study are deposited in the NCBI GEO database with accession number GSE74430.

**How to cite this article:** Luo, G.-Z. *et al*. Characterization of eukaryotic DNA *N*^6^-methyladenine by a highly sensitive restriction enzyme-assisted sequencing. *Nat. Commun.* 7:11301 doi: 10.1038/ncomms11301 (2016).

## Supplementary Material

Supplementary Information Supplementary Figures 1-4 and Supplementary Table 1
