# Supplementary material for: Characterization of eukaryotic DNA N6-methyladenine by a highly sensitive restriction enzyme-assisted sequencing
Source: Nat Commun. 2016 Apr 15;7:11301. doi: 10.1038/ncomms11301 (PMC4835550; doi:10.1038/ncomms11301)
Supplement: Supplementary Information — Supplementary Figures 1-4 and Supplementary Table 1 [file ncomms11301-s1.pdf]

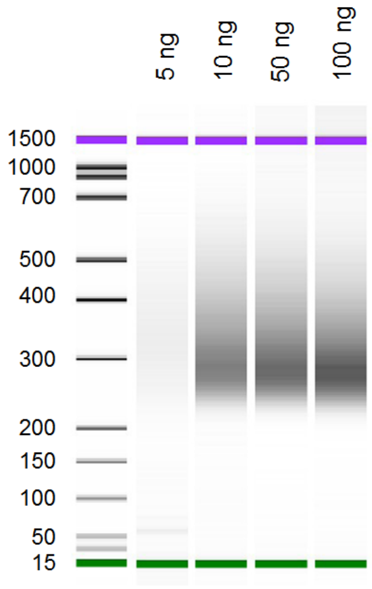

Supplementary Figure 1. Bioanalyzer analysis for DA-6mA-seq library. Standard Illumina libraries (NEBNext DNA library kit) were constructed from 5 ng, 10 ng, 50 ng and 100 ng input DNA.

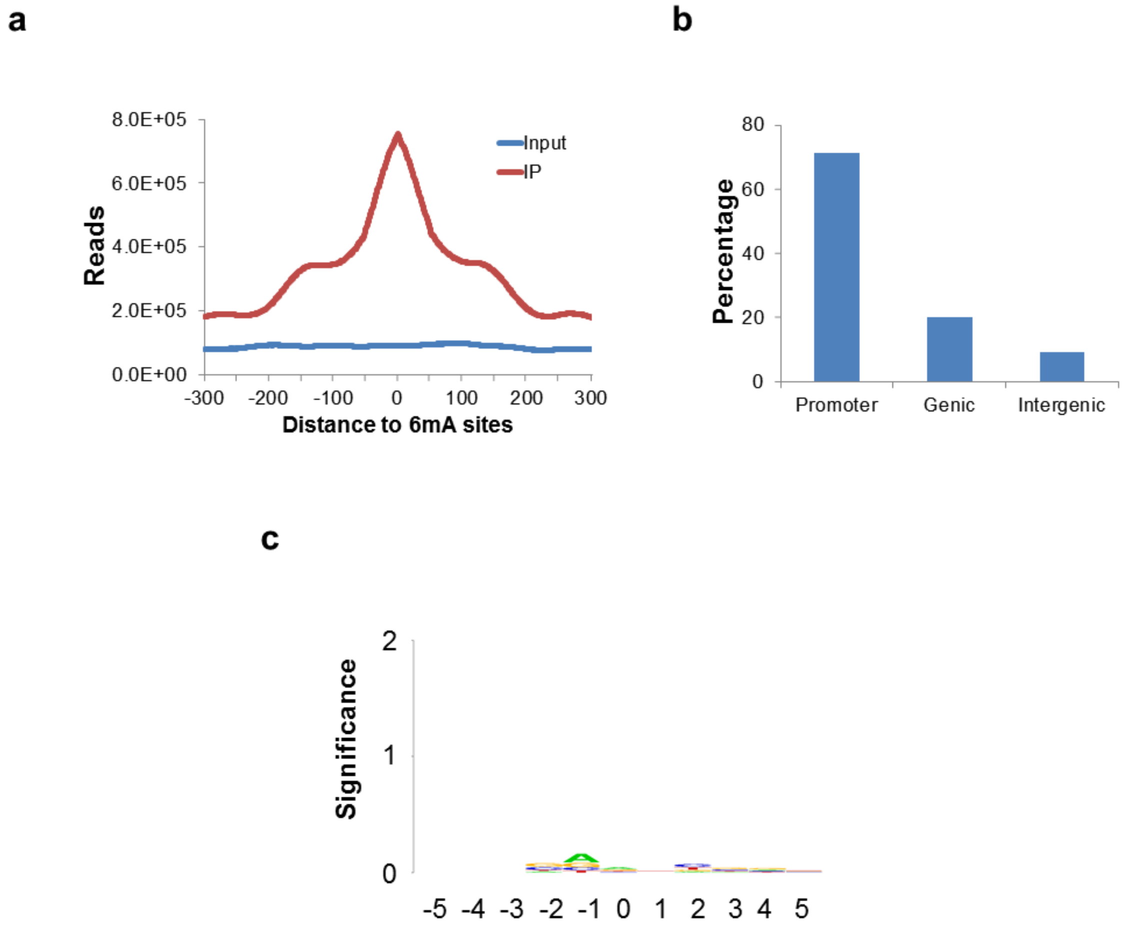

Supplementary Figure 2. C(6mA)TC/G(6mA)TG sites identified by DA-6mA-seq. (a) Reads

pulled down by using anti-6mA immunoprecipitation enriched at C(6mA)TC/G(6mA)TG sites. (b) C(6mA)TC/G(6mA)TG sites are enriched at promoter regions. (c) Sequence preference of 6mA sites besides GATC/CATC/GATG context. Sequence logo is generated by WebLogo. The height represents the significance of enrichment of each nucleotide at that position.

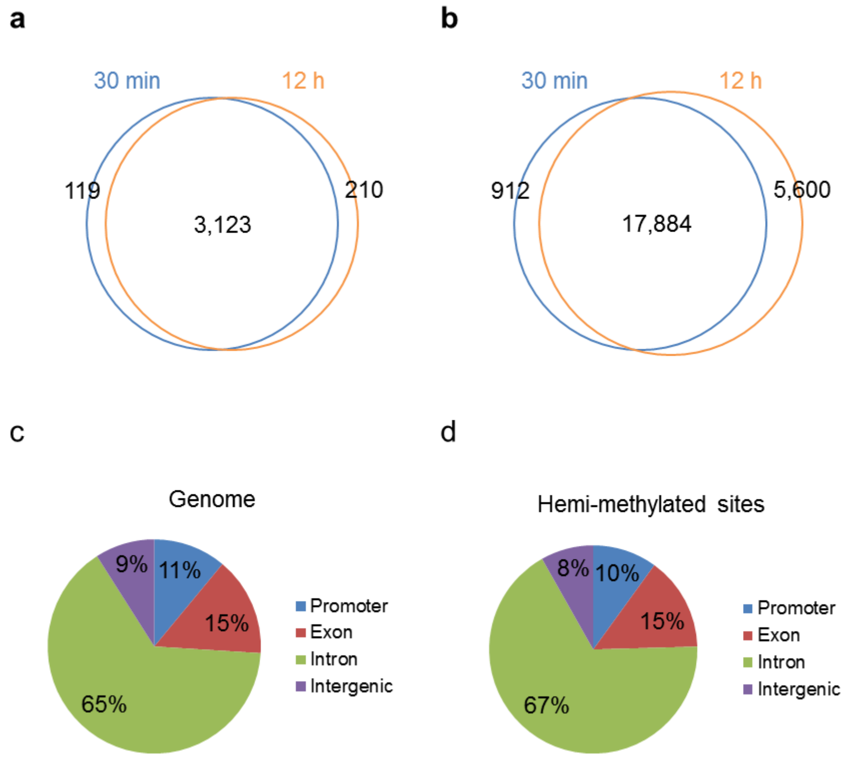

Supplementary Figure 3. DA-6mA-seq detected both fully- and hemi-methylated 6mA sites in *Chlamydomonas*. (a) 6mA sites associated with GATC. The blue circles represent the library treated with DpnI for 30 minutes. The orange circles represent the library treated with DpnI for 12 hours (overnight). (b) 6mA sites associated with CATC/GATG. (c) Compositions of promoters, exons, introns and intergenic regions across the genome of *Chlamydomonas*. (d) Distribution of potential hemi-methylated sites at each genomic region. Hemi-methylated 6mA sites appear to be randomly distributed in the genome of *Chlamydomonas*.

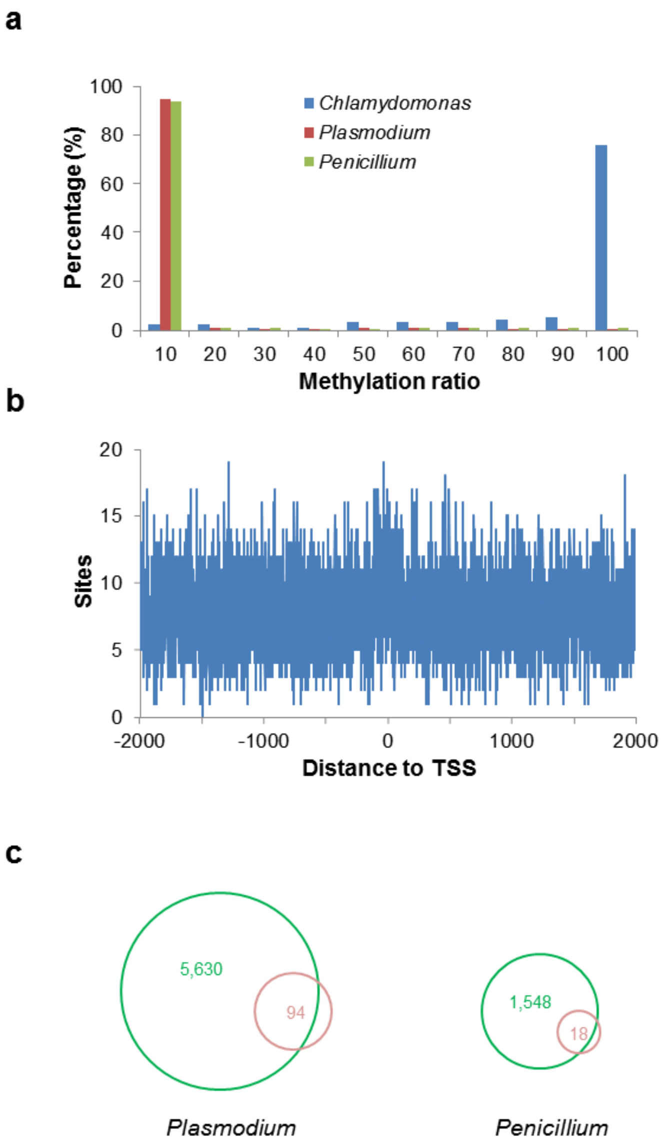

Supplementary Figure 4. Analysis of 6mA in *Plasmodium* and *Penicillium*. (A) Most 6mA sites are almost completely methylated in the genome of *Chlamydomonas*, whereas most 6mA sites in the genomes of *Plasmodium* and *Penicillium* are partially-methylated with the methylation level lower than 10%. (B) The distribution of 6mA sites in *Plasmodium* genome does not show any correlation with gene context. (C) DA-6mA-seq is more sensitive and identifies much more low-methylation-ratio 6mA sites, whereas the previous DpnII-assisted approach detects very limited low-abundance 6mA sites. Left panel: *Plasmodium*; right panel: *Penicillium*.

Supplementary Table 1. The double-stranded DNA probes used in DpnI digestion assay. The methylated adenine is highlighted in red. “F-” represents fully-methylated motif; “H-” represents hemi-methylated motif.

GATC	5'-GCACGCTGCCTCGCCGGGCTTGCTCGGATCTCG-FAM-3'<br>3'-CGTGCGACGGAGCGGCCCGAACGAGCCTAGAGC-5'
F-GATC	5'-GCACGCTGCCTCGCCGGGCTTGCTCGGATCTCG-FAM-3'<br>3'-CGTGCGACGGAGCGGCCCGAACGAGCCTAGAGC-5'
H-GATC	5'-GCACGCTGCCTCGCCGGGCTTGCTCGGATCTCG-FAM-3'<br>3'-CGTGCGACGGAGCGGCCCGAACGAGCCTAGAGC-5'
CATC	5'-GCACGCTGCCTCGCCGGGCTTGCTCGCATCTCG-FAM-3'<br>3'-CGTGCGACGGAGCGGCCCGAACGAGCGTAGAGC-5'
F-CATC	5'-GCACGCTGCCTCGCCGGGCTTGCTCGCATCTCG-FAM-3'<br>3'-CGTGCGACGGAGCGGCCCGAACGAGCGTAGAGC-5'
H-CATC	5'-GCACGCTGCCTCGCCGGGCTTGCTCGCATCTCG-FAM-3'<br>3'-CGTGCGACGGAGCGGCCCGAACGAGCGTAGAGC-5'
